# Total hip arthroplasty for ankylosed hip in young patients: a report of two cases using anterior and posterior approaches

**DOI:** 10.1097/RC9.0000000000000778

**Published:** 2026-08-20

**Authors:** Bagus Wibowo Soetojo, Mohammad Nata Ardiansyah, Bestya Presidiana, Jifaldi Afrian Maharaja Dinda Sedar, Muhammad Zaim Chilmi

**Affiliations:** a Department of Orthopaedic and Traumatology, Airlangga University Hospital, Surabaya, Indonesia; b Slamet Martodirdjo Regional General Hospital, Pamekasan, Indonesia; c Department of Cardiology, Dr. Soetomo General Academic Hospital, Surabaya, Indonesia; d Doctoral Program of Medical Science, Faculty of Medicine, Universitas Airlangga, Surabaya, Indonesia; e Department of Orthopedics and Traumatology, Dr. Soetomo General Academic Hospital, Surabaya, Indonesia; f Department of Orthopedics and Traumatology, Faculty of Medicine, Universitas Airlangga, Surabaya, Indonesia

**Keywords:** ankylosing hip, ankylosing spondylitis, approach hip arthroplasty, hip arthroplasty, joint ankylosis, total hip replacement

## Abstract

**Introduction and importance:**

Total hip arthroplasty (THA) in ankylosed hips is technically challenging due to distorted anatomy, loss of joint space, and difficulty with surgical exposure. The optimal surgical approach remains controversial, particularly in young patients with severe deformities.

**Presentation of case:**

We report two young patients with hip ankylosis who underwent THA using different approaches based on deformity characteristics. A 20-year-old female with bilateral hip ankylosis in external rotation underwent THA via the direct anterior approach, with the Harris Hip Score improving from 0.39% to 53.4%. A 14-year-old male with a fixed flexion-adduction deformity and limb shortening underwent THA via the posterior approach, with the Harris Hip Score improving from 45.35% to 85.25%.

**Clinical discussion:**

These cases demonstrate that the selection of surgical approach should be individualized based on the deformity pattern and intraoperative exposure requirements. The anterior approach may be advantageous for selected patients, whereas the posterior approach provides better visualization in complex deformities.

**Conclusion:**

THA can significantly improve function and quality of life in young patients with ankylosed hips. Both anterior and posterior approaches can achieve favorable outcomes when selected based on deformity characteristics. Careful preoperative assessment and individualized approach selection are essential to optimize functional outcomes.

## Background

Hip ankylosis is characterized by severe restriction or complete loss of hip joint motion due to fibrous or bony fusion of the joint. It may result from inflammatory, traumatic, degenerative, infectious, or iatrogenic causes. In ankylosing spondylitis (AS), progressive inflammation may lead to cartilage destruction, periarticular ossification, and eventual joint fusion, resulting in complex deformity patterns such as adduction–internal rotation or abduction–external rotation^[^1,2^]^.

HIGHLIGHTS

- THA for ankylosed hips in ankylosing spondylitis requires individualized selection of the surgical approach.
- The direct anterior approach provided excellent functional recovery in cases of bilateral external rotation ankylosis.
- The posterior approach facilitated the correction of a severe flexion–adduction deformity with a limb-length discrepancy.
- Tailored surgical planning can achieve favorable functional outcomes in complex ankylosed hips.

Total hip arthroplasty (THA) is a well-established procedure for alleviating pain and restoring mobility in patients with advanced hip disorders. However, its application in ankylosed hips, particularly in individuals with ankylosing spondylitis (AS), remains technically demanding and clinically controversial. In these patients, progressive inflammation frequently results in hip joint fusion, leading to complex deformity patterns such as adduction–internal rotation or abduction–external rotation. These variations pose distinct challenges for surgical exposure, osteotomy, and implant positioning^[^1^]^. The absence of standardized guidelines further complicates decision-making, often requiring surgeons to rely on individual experience and institutional practices^[^2^]^. Comprehensive preoperative assessment and an understanding of AS pathophysiology are essential for planning.

We present two cases of THA in ankylosed hips, highlighting the importance of individualized surgical strategies tailored to specific anatomical characteristics and deformities to achieve optimal functional outcomes. This case report has been prepared in accordance with the SCARE checklist^[^3^]^.

## Case report

### Case 1

**Figure 1.**
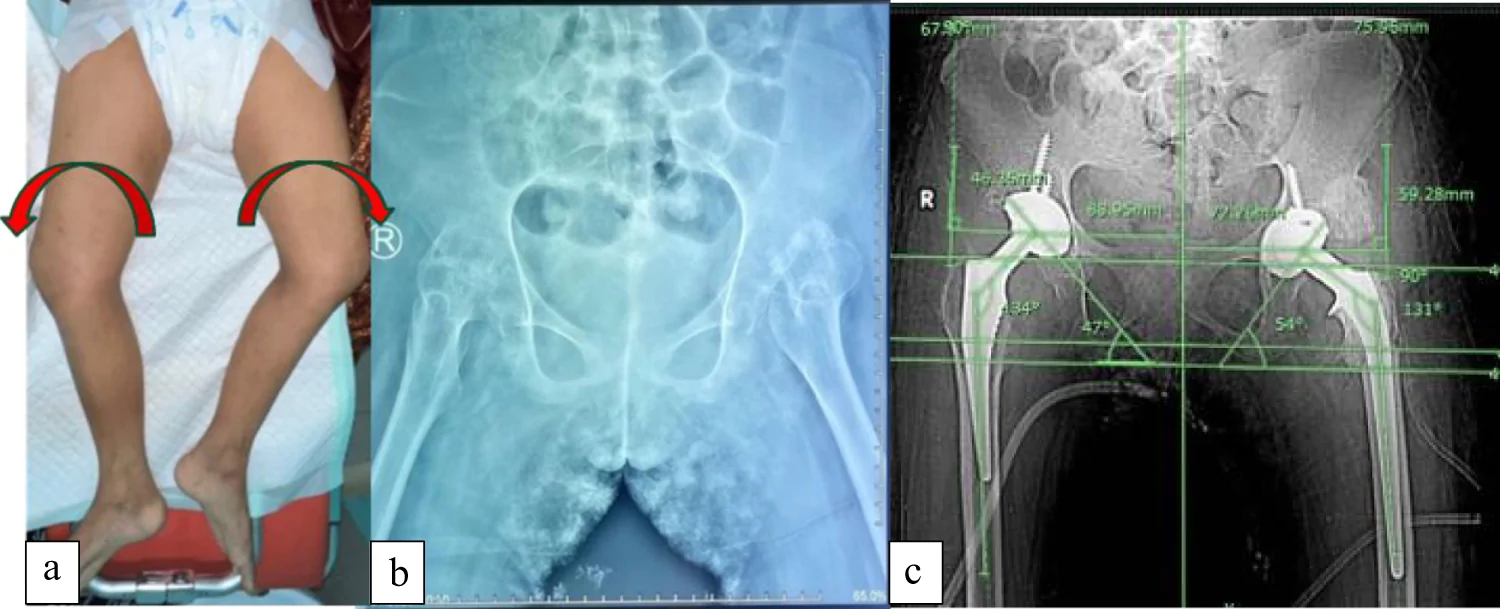
Preoperative and postoperative clinical and radiological images of the hips: (a) clinical photographs of the right and left hips in external rotation; (b) radiographs of the pelvis before surgery; (c) radiographs of the pelvis after surgery.

A 20-year-old female presented with bilateral hip pain and progressive stiffness for two years following a fall from height. After the injury, she was taken to a traditional bone setter and subsequently became unable to walk. She had a history of aplastic anemia and was receiving methylprednisolone. The patient reported severe functional limitation affecting daily activities. Clinical examination revealed bilateral hip ankylosis with fixed external rotation deformity (Fig. 1a). Range of motion was markedly restricted (fixed hip flexion 30°, external rotation 45°, abduction 30°), with a 1 cm leg length discrepancy. The Harris Hip Score (HHS) was 0.39%. Radiographic evaluation confirmed bilateral hip joint ankylosis with anterior dislocation (Fig. 1b). A preoperative pelvic CT scan was not performed; surgical planning was based on clinical examination and plain radiographs.

**Figure 2.**
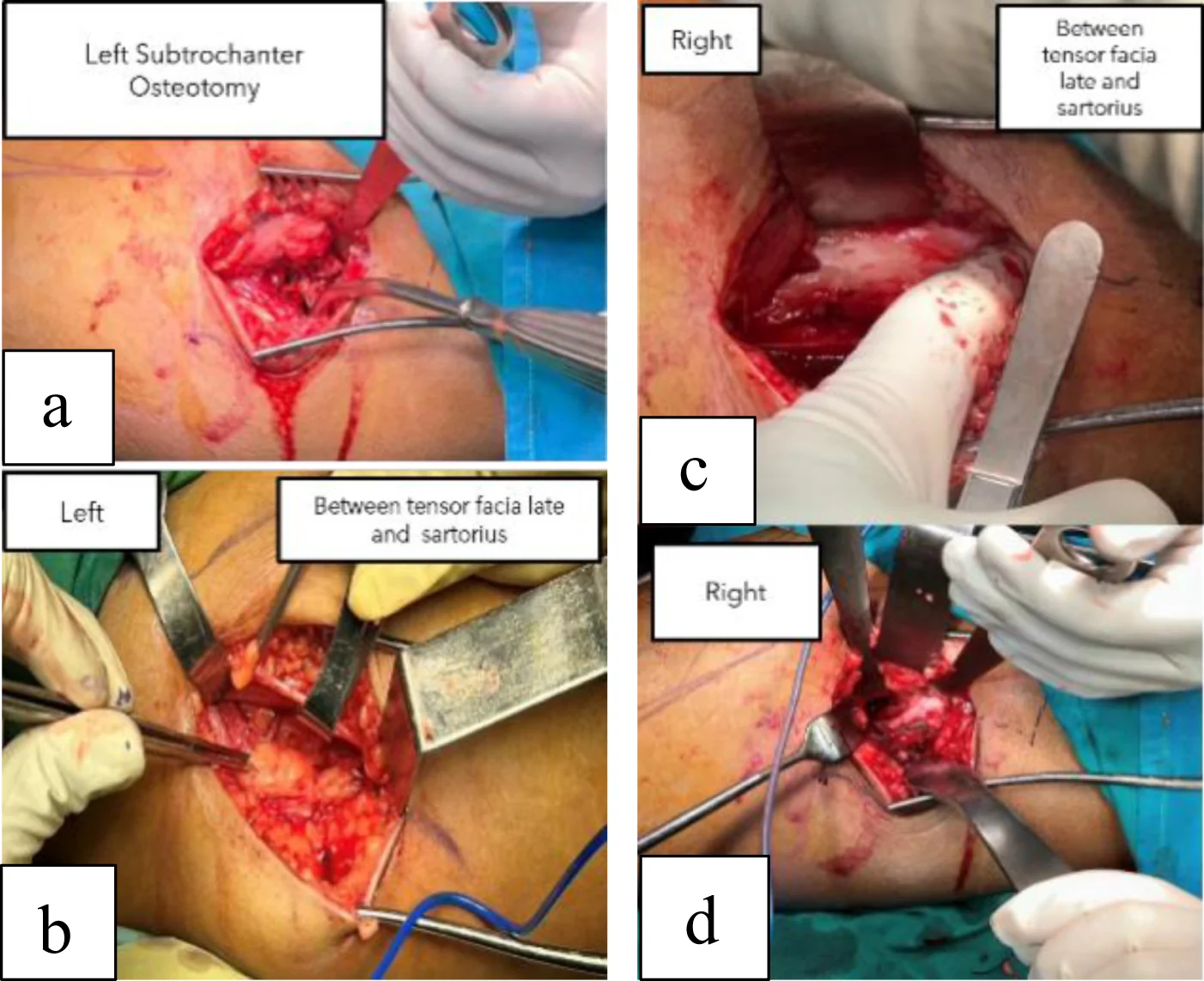
Case 1: During THA procedures on the right and left hips using an anterior approach: (a) left subtrochanteric osteotomy; (b) approach to the femoral neck between the TFL and sartorius; (c) right hip exposure through the intermuscular plane between the tensor fascia lata and sartorius; (d) deeper exposure of the right femoral neck during the anterior approach prior to femoral neck osteotomy. TFL, tensor fascia lata.

The patient underwent total hip arthroplasty under general anesthesia using the direct anterior approach by an experienced orthopaedic surgeon. Intraoperatively, exposure was achieved through the intermuscular plane between the tensor fascia lata (TFL) and sartorius (Fig. 2b). A subtrochanteric osteotomy was performed on the left hip to facilitate mobilization, deformity correction, and surgical exposure (Fig. 2a). In the right hip, exposure through the same intermuscular plane was performed with a standard femoral neck osteotomy, without subtrochanteric osteotomy (Fig. 2c and d). Short-stem and long-stem cementless prostheses were implanted in the right and left hips, respectively. The patient was hospitalized for five days. The patient underwent right hip surgery first, followed by left hip surgery one month later because of national insurance. One month after both procedures had been completed, partial weight-bearing ambulation with a walker was permitted.

Postoperatively, the patient demonstrated substantial improvements in range of movement (ROM), with hip flexion increasing to 120 degrees, external rotation to 50 degrees, abduction to 45 degrees, and a significant improvement in the HHS from 0.39% to 53.4% at 1 year after surgery. These improvements represent a significant enhancement in joint mobility and function compared to the preoperative state. The patient also reported a reduction in pain, improved functionality, and an overall better quality of life. Furthermore, the correction of the leg length discrepancy contributed to enhanced gait and balance, as demonstrated on radiographic (Fig. 1c).

### Case 2

**Figure 3.**
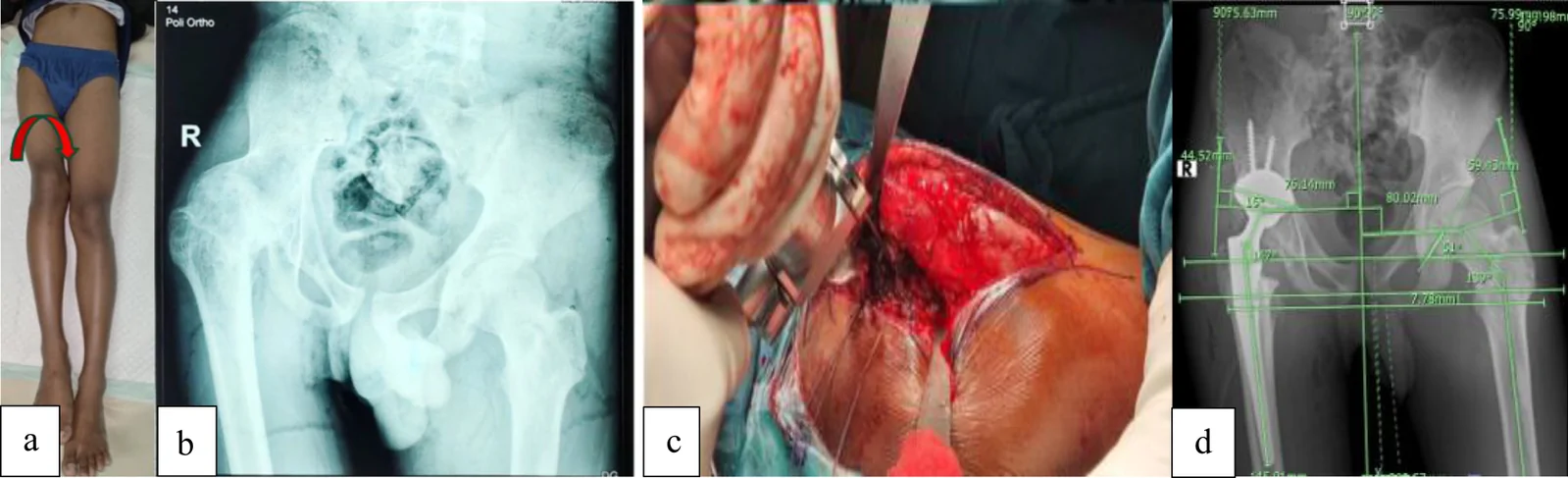
Preoperative, intraoperative, and postoperative clinical and radiographic findings for Case 2. (a) Clinical appearance before surgery, showing right lower-limb deformity, shortening, and internal rotation of the hip. (b) Pelvic radiograph before surgery, showing right hip ankylosis. (c) Intraoperative view during THA through the posterior approach. (d) Pelvic radiograph after surgery.

The second patient was a 14-year-old male who presented with right hip pain and a persistent limp following a motorcycle accident three years earlier. After the injury, he was treated by a traditional bone setter. The patient had no relevant past medical or surgical history and was not taking any regular medications. Following the injury, he developed progressive pain, stiffness, deformity, and shortening of the right lower limb. Recent exacerbations of pain over the last ten days prompted his visit. Physical examination revealed a fixed flexion-adduction and internal rotation deformity of the right hip, shortening of the right lower limb, and muscle atrophy of the right lower extremity. (Fig. 3a). ROM was limited, and leg length discrepancy measured 8 cm and HHS of 45.35%. Pelvic radiographs demonstrated right hip ankylosis with chronic posterior dislocation (Fig. 3b). A preoperative pelvic CT scan was not performed; surgical planning was based on clinical examination and plain radiographs

Given the deformity severity, need for extensive exposure, and history of posterior dislocation, THA under general anesthesia was performed via the posterior approach by an experienced orthopaedic surgeon, providing adequate visualization of the acetabulum and proximal femur for deformity correction and limb-length restoration (Fig. 3c). A short-stem cementless prosthesis was used. The patient was hospitalized for four days. Mobilization with partial weight-bearing with walker was initiated on the first days postoperative.

Postoperative radiographs confirmed satisfactory alignment with a residual 0.7 cm leg length discrepancy (Fig. 3d). At one year, HHS improved from 45.35% to 85.25%, with significant improvement in hip ROM, stability, pain relief, and quality of life, facilitating the patient’s return to normal activities.

## Discussion

Total hip arthroplasty (THA) in ankylosed hips remains technically challenging because of severe stiffness, distorted anatomy, abnormal hip positioning, and difficulty identifying anatomical landmarks. In patients with Ankylosing Spondylitis (AS) or long-standing ankylosis, deformities such as flexion, adduction, abduction, and rotational malalignment complicate surgical exposure, femoral neck osteotomy, acetabular preparation, restoration of the hip centre, and correction of limb-length discrepancy. Careful preoperative assessment of deformity pattern, bone quality, spinopelvic alignment, and functional limitation is therefore essential for surgical planning^[^1,2,4^]^.

The present cases highlight the importance of individualized surgical planning. In the first case, an external rotation deformity allowed the use of the direct anterior approach (DAA), facilitating muscle preservation and early postoperative recovery. In contrast, the second case involved a fixed flexion-adduction deformity with marked limb shortening, requiring a posterior approach to achieve adequate exposure and reconstruction. Both patients experienced substantial functional improvement, consistent with previous reports demonstrating that THA significantly improves pain, mobility, and quality of life in ankylosed hips^[^2,5,6^]^.

The DAA utilizes an intermuscular and internervous plane, minimizing muscle disruption and potentially reducing postoperative pain, dislocation risk, and time to functional recovery^[^5,7^]^. However, it has a steep learning curve and may be technically demanding in patients with severe deformities^[^5–7^]^. Conversely, the posterior approach provides excellent visualization of both the acetabulum and proximal femur, making it particularly useful in complex reconstructions and severe deformities. Although the posterior approach has historically been associated with a higher risk of dislocation, meticulous soft-tissue repair and accurate component positioning can substantially reduce this risk^[^6,7^]^. In ankylosed hips, the surgical approach should be selected according to the deformity pattern, joint fusion, and required exposure. The direct anterior approach may reduce soft-tissue injury but can be technically difficult in severe deformities. In contrast, the posterior approach provides wider exposure and may be more suitable for fixed flexion-adduction deformity, marked limb shortening, or posterior displacement. Additional osteotomy may be required to improve exposure and correct the deformity. Regardless of the approach, careful acetabular identification, component positioning, neurovascular protection, and limb-length correction are essential to minimize complications^[^5,7^]^.

Previous studies have reported favorable outcomes of THA in ankylosed hips and AS. Siavashi *et al* demonstrated significant improvements in HHS and hip range of motion^[^6^]^, while Saglam *et al* reported satisfactory mid-term functional and radiological outcomes^[^4^]^. Long-term data from Bukowski *et al* showed excellent implant survivorship, although continued surveillance for revision surgery remains necessary^[^8^]^.Favorable outcomes have also been reported in young patients with severe hip disorders. Megremis et al. reported successful THA in a 12-year-old girl with protrusio acetabuli secondary to idiopathic chondrolysis. Her Harris Hip Score improved from 38.45 before surgery to 97 at the six-year follow-up. Falgiano et al. reported staged bilateral THA using a posterior approach and modular dual-mobility implants in a 33-year-old man with bilateral bony hip ankylosis. At one year, the patient was able to walk without an assistive device, and his Harris Hip Score improved from 29 to 90. These reports suggest that THA can provide good outcomes in young patients with severe hip stiffness or ankylosis when the surgical approach and implant are selected according to the deformity^[^9–11^]^. Behesti Fard et al. reported a successful single stage bilateral hip conversion arthroplasty via the direct anterior approach in a 26-year-old Persian male with a history of AS and severe kyphoscoliosis, leading to bilateral hip fusion and immobility 12. Current evidence suggests that neither anterior nor posterior approaches are universally superior; rather, approach selection should be guided by deformity characteristics, anatomical considerations, and surgeon expertise (Table 1).

**Table 1:** Current evidence on similar cases of ankylosis.

Author, year	Country	Patients/hips	Cause of ankylosis	Surgical approach	Follow-up	Main outcome
Siavashi *et al*., 2014	Iran	77 patients	Mixed etiologies	Posterior in 55 patients; lateral in 22 patients	12 months	Mean HHS improved from 48.53 to 88.22
Bukowski *et al*., 2021	United States	219 patients/309 hips	Ankylosing spondylitis	Direct anterior approach	Mean 16 years	HHS improved from 51 to 76; revision incidence 17.5% at 20 years
Falgiano *et al*., 2024	United States	33-year-old male/bilateral hips	Suspected ankylosing spondylitis with bilateral bony hip ankylosis	Staged bilateral posterior THA using modular dual-mobility implants	1 year	HHS improved from 29 to 90; the patient walked without an assistive device
Beheshti Fard *et al*., 2023	Iran	1 patients 2 hips	Ankylosing spondylitis, bilateral hip fusion, severe kyphoscoliosis	Direct anterior approach single stage bilateral THA	12 months	Good outcome (no quality score reported)
Case 1	Indonesia	20-year-old female/bilateral hips	Post-traumatic	Direct anterior approach	1 year	HHS improved from 0.39 to 53.4
Case 2	Indonesia	14-year-old male/right hip	Post-traumatic	Posterior approach	1 year	HHS improved from 45.35 to 85.25

Potential complications of THA in ankylosed hips include intraoperative fracture, neurovascular injury, dislocation, heterotopic ossification, aseptic loosening, and revision surgery. Comprehensive preoperative planning, appropriate implant selection, meticulous surgical technique, and structured rehabilitation remain critical for optimizing outcomes^[^13–15^]^.

## Limitations

This report has several limitations. First, the inclusion of only two cases precludes comparative analysis and prevents drawing conclusions regarding the superiority of one surgical approach over another. Second, the relatively short follow-up period limits the assessment of long-term implant survivorship, late complications, and revision risk. Third, both patients were young, making long-term monitoring particularly important.

Despite these limitations, both cases demonstrated substantial functional improvement and enhanced quality of life following THA, supporting the value of individualized surgical planning in managing ankylosed hips.

## Conclusion

THA is an effective but technically demanding treatment for ankylosed hips, particularly in patients with ankylosing spondylitis. These cases demonstrate that both the direct anterior and posterior approaches can achieve favorable outcomes when selected according to deformity characteristics and surgical requirements. Careful preoperative assessment and individualized approach selection remain essential to optimize functional recovery and quality of life.

## Data Availability

The data that support the findings of this study are available from the corresponding author on reasonable request.

## Prior presentation

The authors confirm it is not under consideration elsewhere.
